# Fatty acid synthase regulates estrogen receptor-α signaling in breast cancer cells

**DOI:** 10.1038/oncsis.2017.4

**Published:** 2017-02-27

**Authors:** J A Menendez, R Lupu

**Affiliations:** 1 Program Against Cancer Therapeutic Resistance (ProCURE), Metabolism and Cancer Group, Catalan Institute of Oncology, Girona, Catalonia, Spain; 2 Molecular Oncology Group, Girona Biomedical Research Institute (IDIBGI), Girona, Catalonia, Spain; 3 Mayo Clinic, Division of Experimental Pathology, Department of Laboratory Medicine and Pathology, Rochester, MN, USA; 4 Mayo Clinic Cancer Center, Rochester, MN, USA

## Abstract

Fatty acid synthase (FASN), the key enzyme for endogenous synthesis of fatty acids, is overexpressed and hyperactivated in a biologically aggressive subset of sex steroid-related tumors, including breast carcinomas. Using pharmacological and genetic approaches, we assessed the molecular relationship between FASN signaling and estrogen receptor alpha (ERα) signaling in breast cancer. The small compound C75, a synthetic slow-binding inhibitor of FASN activity, induced a dramatic augmentation of estradiol (E_2_)-stimulated, ERα-driven transcription. FASN and ERα were both necessary for the synergistic activation of ERα transcriptional activity that occurred following co-exposure to C75 and E_2_: first, knockdown of FASN expression using RNAi (RNA interference) drastically lowered (>100 fold) the amount of E_2_ required for optimal activation of ERα-mediated transcriptional activity; second, FASN blockade synergistically increased E_2_-stimulated ERα-mediated transcriptional activity in ERα-negative breast cancer cells stably transfected with ERα, but not in ERα-negative parental cells. Non-genomic, E_2_-regulated cross-talk between the ERα and MAPK pathways participated in these phenomena. Thus, treatment with the pure antiestrogen ICI 182 780 or the potent and specific inhibitor of MEK/ERK, U0126, was sufficient to abolish the synergistic nature of the interaction between FASN blockade and E_2_-stimulated ERα transactivation. FASN inhibition suppressed E_2_-stimulated breast cancer cell proliferation and anchorage-independent colony formation while promoting the reduction of ERα protein. FASN blockade resulted in the increased expression and nuclear accumulation of the cyclin-dependent kinase inhibitors p21^WAF1/CIP1^ and p27^Kip1^, two critical mediators of the therapeutic effects of antiestrogen in breast cancer, while inactivating AKT, a key mediator of E_2_-promoted anchorage-independent growth. The ability of FASN to regulate E_2_/ERα signaling may represent a promising strategy for anticancer treatment involving a new generation of FASN inhibitors.

## Introduction

The early and near universal upregulation of the lipogenic enzyme fatty acid synthase (FASN) in most human cancers together with its association with poor clinical outcome support the hypothesis that endogenous fatty acid metabolism is involved in the development, maintenance and enhancement of the malignant phenotype.^1, 2, 3, 4, 5, 6, 7, 8, 9^ However, the increased FASN expression and catalytic activity in tumor cells seem to be part of a more general change in the genetic program controlling lipogenesis as evidenced by the concomitant increase in other enzymes of the same lipogenic pathway.^10, 11^ The question therefore arises as to whether activation of FASN actively contributes to the cancer phenotype or is merely a manifestation of an early and common dysregulation of upstream signaling pathways in neoplastic cells. Indeed, a currently favored hypothesis posits an epigenetic basis of increased FASN expression in cancer cells and suggests that changes in upstream regulatory circuits (for example, hormones/growth factors and their receptors → lipogenic transcription factors→lipogenic genes) underlie, at least in part, this phenomenon.^11^

Previous research in our laboratory demonstrated that pharmacological inhibition of FASN activity markedly reduces HER2 oncogene expression in cancer cells.^11, 12, 13^ RNA interference (RNAi)-mediated silencing of FASN also represses HER2 expression. Conversely, inhibition of HER2 induces downregulation of FASN,^12^ revealing a bi-directional molecular relationship between HER2 and FASN. These findings highlighted a previously unrecognized signaling pathway emerging from endogenous fatty acid metabolism, an anabolic-energy-storage pathway largely considered of minor importance in humans. Moreover, these data bolster the premise that tumor-associated FASN is not only necessary to integrate a number of signaling pathways that regulate metabolism, proliferation, and survival in cancer cells, but also has an active role in carcinogenesis by regulating proteins involved in malignant transformation.^12, 13, 14^ Unraveling the molecular interplay between well-characterized cancer-related networks and FASN-dependent neoplastic lipogenesis is a major challenge that the cancer field is only now beginning to realize.

Although the precise mechanism underlying FASN overexpression in tumors is still unclear, it has been shown that estradiol (E_2_), progesterone, and androgens can regulate FASN expression in hormonally responsive tumors. Thus, FASN expression is part of the E_2_-driven cellular response that leads to proliferation in hormone-dependent endometrial carcinoma cells, and it is associated with higher endometrial tumor grades.^15^ Further, E_2_, progesterone, and synthetic progestins also stimulate cell growth and concomitant FASN expression in hormone-dependent human breast cancer cells.^16, 17, 18^ The identification of a novel FASN/estrogen receptor alpha (ERα) fusion transcript expressed in a variety of human cancer cell lines further suggests a close linkage between FASN and the ERα signaling pathway.^19^ We here aimed to characterize the involvement of FASN-catalyzed endogenous fatty acid biosynthesis on E_2_-independent and -dependent ERα signaling in human breast cancer cells. We present evidence herein that the aberrant lipogenic activity of tumor-associated FASN regulates the response of breast cancer cells to E_2_-stimulated ERα signaling.

## Results

### Pharmacological inhibition of FASN activity synergistically enhances E_2_-stimulated ERα-driven transcriptional activity

**Figure 1:**
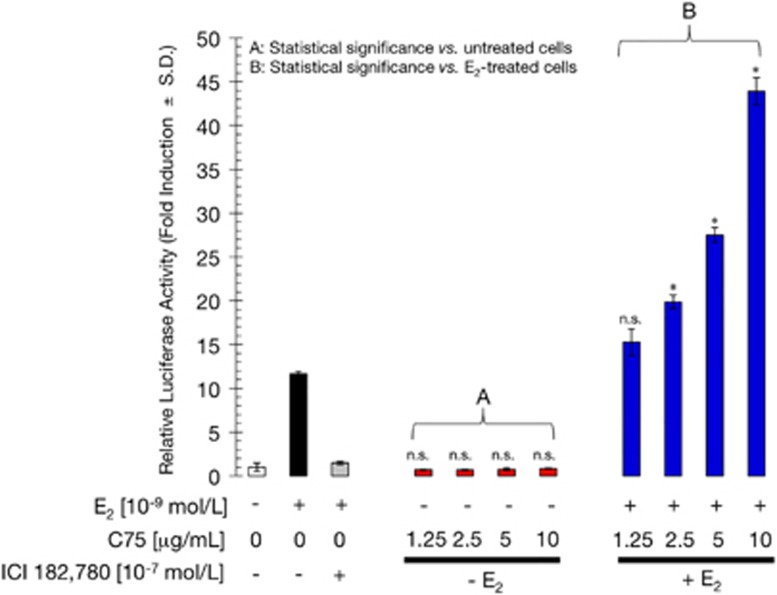
FASN inhibition synergistically enhances E_2_-dependent ERα-driven transcriptional activity in MCF-7 cells. MCF-7 cells were transiently co-transfected with an ERE-luciferase reporter and pRL/CMV. Transfected cells were incubated for 24 h with vehicle (control), E_2_, ICI 182 780, or C75 individually or in combination as indicated, and cell extracts were assayed for luciferase activity. Data represent mean±s.d. (*n*=4). Non-significant (NS) differences (*P*>0.05) were identified by ANOVA followed by Scheffé's multiple contrasts; *P<0.05 compared with control cells by ANOVA followed by Scheffé's multiple contrasts. (**a**) Control cells in culture medium only. (**b**) Control cells in medium supplemented with 10^−9^ mol/l E_2_.

To evaluate the effects of FASN inhibition on ERα transactivation and E_2_ responsiveness, ERα-positive MCF-7 breast cancer cells were co-transfected with a luciferase reporter gene linked to an estrogen response element (ERE-Luciferase), and an internal control vector (pRL-CMV). Transfected cells were then evaluated for changes in the levels of basal (E_2_-independent) and E_2_-dependent reporter activity in the presence of increasing concentrations of the chemical FASN inhibitor C75.^20^ When FASN-inhibited MCF-7 cells were compared with untreated cells, E_2_-independent ERα-driven transcriptional activity was unchanged by FASN blockade (Figure 1). Next, transfected MCF-7 cells were treated with a combination of E_2_ and C75 to determine whether their actions were additive, synergistic, or antagonistic. As a single agent, E_2_ (10^−9^ mol/l) induced a ∼12-fold increase in luciferase activity relative to basal levels in untreated cells. As expected, co-treatment with the pure antiestrogen ICI 182 780 (10^−7^ mol/l) antagonized E_2_-induced luciferase activity. Interestingly, co-exposure to E_2_ and C75 resulted in a dose-dependent increase (up to ∼44-fold) in ERE reporter activity, which represents a synergistic ∼4-fold increase in ERα-dependent transcriptional activity relative to the activity found in E_2_-stimulated MCF-7 cells (Figure 1). These results demonstrate that pharmacological blockade of FASN activity hypersensitizes breast cancer cells to E_2_-dependent ERα transactivation.

### C75-regulated ERα transactivation involves ERα and FASN

**Figure 2:**
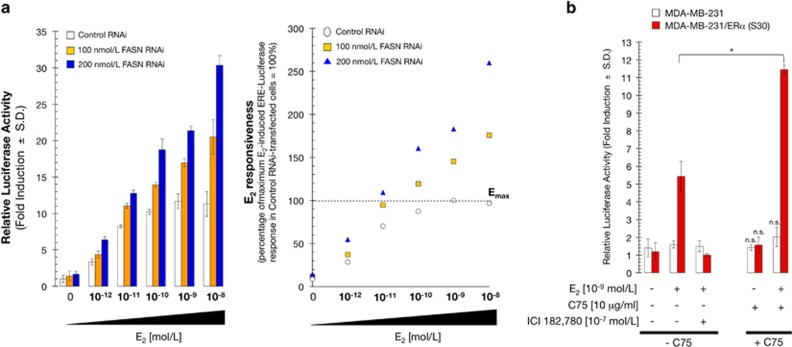
Stimulation of ERα transcriptional activity by pharmacological or molecular inhibition of FASN. (**a**) Left: FASN-depleted MCF-7 cells were transiently transfected with ERE-luciferase reporter and pRL/CMV and then exposed for 24 h to medium containing E_2_ as indicated, harvested and assayed for luciferase activity. The data shown represent mean±s.d. (*n*=4). Right: Percentage stimulation of E_2_-induced ERE-luciferase activity was obtained from curve-fits of individual concentration-response curves (third-order polynomial, with all *R* values>0.95) based on comparison to the optimal activity obtained from E_2_-stimulated MCF-7 cells transfected with control RNAi (set at 100%). (**b**) ERα-negative MDA-MB-231 cells and MDA-MB-231 cells stably transfected with ERα (S30 cells) were transiently co-transfected with ERE-luciferase reporter and pRL/CMV and then incubated for 24 h with vehicle (control), E_2_, ICI 182 780, or C75, individually or in combination. Cell extracts were assayed for luciferase activity and the data represent mean±s.d. (*n*=4); non-significant (NS) differences (*P*>0.05) were identified by ANOVA followed by Scheffé's multiple contrasts; *P<0.05 compared with control cells by ANOVA followed by Scheffé's multiple contrasts.

To rule out off-target effects of the FASN inhibitor C75, FASN gene expression was silenced using RNAi. We predicted that if FASN was responsible for C75-induced hypersensitivity to E_2_, downregulation of FASN would lead to enhanced ERα-driven transcriptional activity following stimulation with E_2_. Western blot analysis of MCF-7 cells demonstrated that transfection of 200 nmol/l FASN RNAi, but not an equivalent concentration of nonspecific RNAi, severely suppressed FASN expression and activity (Supplementary Figure 1).^21^ FASN silencing resulted in a 2–3-fold increase in E_2_-stimulated reporter activity relative to control cells (Figure 2a, left). Thus, FASN-depleted cells showed strong ERE transcriptional activity at much lower E_2_ concentrations (4 × 10^−11^ mol/l E_2_ at 100 nmol/l FASN RNAi and 6 × 10^−12^ mol/l E_2_ at 200 nmol/l FASN RNAi) than control cells (10^−9^ mol/l E_2_; Figure 2a, right). These data indicate that RNAi-mediated silencing of FASN markedly reduces the E_2_ requirement for ERα transactivation, further supporting the notion that C75 likely exerts its sensitizing effects on E_2_-stimulated ERα-driven transcriptional activity through its FASN target. More importantly, these results reveal that FASN activity is a novel regulator of ERα signaling in hormone-responsive breast cancer cells.

The ERα-negative breast cancer cell line MDA-MB-231 was then used to confirm that ERα is required for C75-induced hypersensitivity to E_2_. In the absence of ERα (that is, wild-type MDA-MB-231 cells), C75 had no effect on ERE transcriptional activity in the absence or presence of E_2_ (Figure 2b). By contrast, in MDA-MB-231 cells stably expressing wild-type ERα (S30 cells),^22, 23^ C75 and E_2_ co-treatment synergistically stimulated ERE transcriptional activity. This synergism between E_2_ and C75 was abolished by ICI 182 780, demonstrating that the stimulatory effects of C75-induced inhibition of FASN activity on ERα transactivation require ERα.

### FASN inhibition enhances E_2_-stimulated ERα-driven transcriptional activity through the activation of non-genomic ERα/MAPK cross-talk

**Figure 3:**
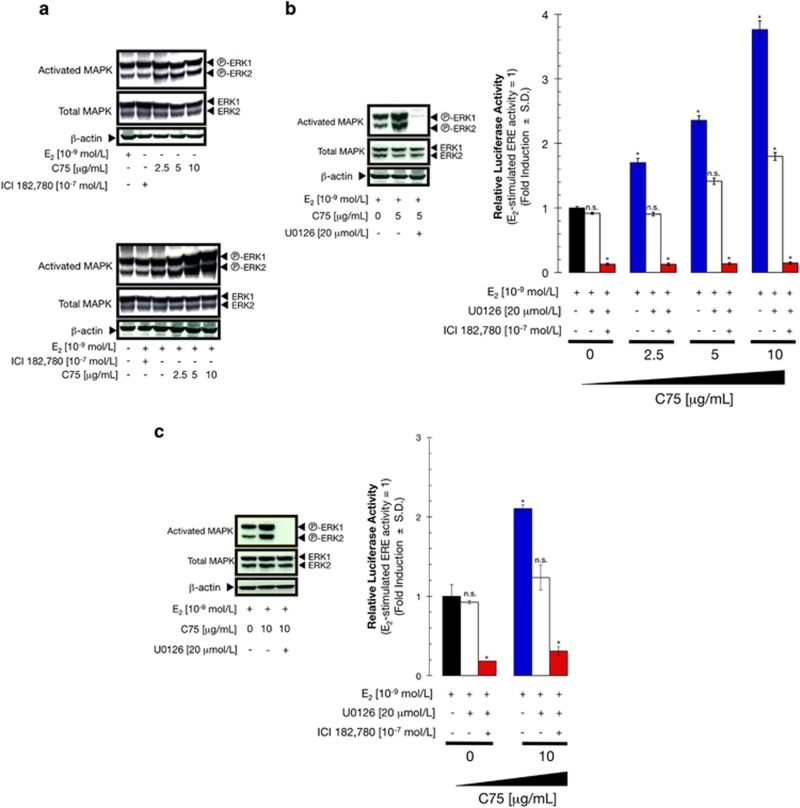
C75 modulates non-genomic cross-talk between ERα and MAPK. (**a**) C75 treatment activates MAPK. MCF-7 cells were lysed following treatment with C75 with (top panel) or without (bottom panel) E_2_ for 48 h, and MAPK activation was determined by western blotting with a phospho-ERK1/2 antibody. ICI 182 780 was used as a negative control. The blot was stripped and re-probed with antibodies for total ERK1/2 protein and β-actin to assess loading and transfer efficiency. (**b**, **c**) The MEK1/2 inhibitor U0126 suppresses C75-induced MAPK activation. Protein was isolated from MCF-7 cells (**b**) and S30 cells (**c**) following treatment with C75 and E_2_ for 48 h (Figure 3c) and analyzed as indicated. MCF-7 (**b**) and S30 (**c**) cells were transiently co-transfected with ERE-luciferase reporter and pRL/CMV. Transfected cells were incubated for 24 h with vehicle, E_2_, ICI 182 780, C75 or U0126, individually or in combination as specified. Cell extracts were assayed for luciferase activity. Data represent mean±s.d. (*n*=4); non-significant (NS) differences (*P*>0.05) were identified by ANOVA followed by Scheffé's multiple contrasts; *P<0.05 compared with control cells (in medium supplemented with 10^−9^ mol/l E_2_) by ANOVA followed by Scheffé's multiple contrasts.

The synergistic nature of the interaction between E_2_ and C75 was consistent with both compounds activating ERα-mediated transcriptional activity through different molecular mechanisms. We therefore investigated whether FASN inhibition modulated the MAPK signaling pathway, which has been repeatedly shown to sensitize breast cancer cells to E_2_.^24, 25, 26, 27, 28, 29, 30, 31^ Under E_2_-depleted conditions, C75 treatment marginally activated MAPK (Figure 3a, top panel). The levels of activated MAPK increased slightly following treatment with E_2_, and this activation was reversed by co-treatment with ICI 182 780 (Figure 3a, bottom panel). Remarkably, C75 induced a concentration-dependent increase of activated MAPK in E_2_-stimulated MCF-7 cells (Figure 3a, bottom panel). Western blot analyses similarly revealed that the potent and specific MEK1/MEK2 inhibitor U0126 suppressed C75-induced activation of MAPK in E_2_-stimulated MCF-7 and S30 cells (Figure 3b, left panel). Together, these results demonstrate that suppression of FASN-driven endogenous lipogenesis is a novel upstream event regulating the MEK1/MEK2 → ERK1/ERK2 cascade in breast cancer cells.

To determine if MAPK/ERα cross-talk underlies the C75-induced enhancement of E_2_-stimulated ERα transactivation, we next tested whether blocking MAPK signaling would decrease ERα-driven transcriptional activity. Indeed, treatment with U0126 significantly reversed the stimulatory effect of C75 on ERE-mediated luciferase reporter activity to the level observed by E_2_ stimulation alone (Figure 3b, right panel), whereas co-treatment with U0126 and ICI 182 780 abolished ERE reporter activity induced by E_2_ and C75 co-treatment. Similar results were obtained in S30 cells (Figure 3c). These data imply that activation of MAPK has a key role in C75-enhanced, E_2_-stimulated ERE reporter activity. Furthermore, they strongly suggest that C75 positively regulates E_2_-stimulated ERα transactivation through activation of non-genomic ERα/MAPK cross-talk.

### FASN inhibition markedly enhances E_2_-stimulated ERα transactivation in breast cancer cells exhibiting constitutive MAPK hyperactivation

MCF-7 human breast cancer cells transfected with a full-length cDNA of the HER2 oncogene or treated with ectopic heregulin (HRG; the HER-3/-4 ligand that transactivates HER2) lose E_2_ dependence.^32, 33, 34^ Multiple lines of evidence suggest that differences in E_2_ responsiveness in these models are not attributable to differences in ERα expression levels but rather to the potential interaction of signal transduction elements that link HER2 to ERα. Specifically, unrestrained MAPK signaling seems to have a key role in determining E_2_-dependent ERα activity in the presence of upstream oncogenic stimuli such as HER2 and HRG.^30, 35, 36, 37^

**Figure 4:**
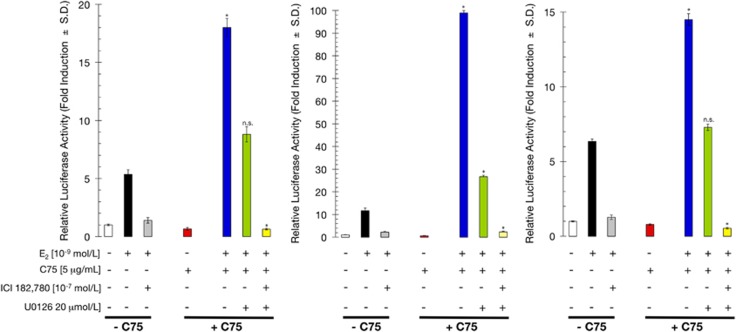
FASN inhibition significantly enhances E_2_-dependent ERα transcriptional activity in MCF-7/HER2-18, BT-474 and MCF-7/HRG cells. BT-474 (left), MCF-7/HER2-18 (middle), and MCF-7/HRG (right) cells were transiently co-transfected with an ERE-luciferase reporter and pRL/CMV. Transfected cells were incubated for 24 h with vehicle (control), E_2_, ICI 182 780, C75 or U0126, individually or in combination as indicated. Cell extracts were assayed for luciferase activity and the data represent mean±s.d. (*n*=4). Non-significant (NS) differences (*P*>0.05) were identified by ANOVA followed by Scheffé's multiple contrasts; *P<0.05 compared with control cells (in medium supplemented with 10^−9^ mol/l E_2_) by ANOVA followed by Scheffé's multiple contrasts.

Based on our results presented so far, we predicted that breast cancer cells with constitutive MAPK hyperactivation would exhibit a more pronounced sensitivity to E_2_-stimulated, ERα-mediated transcriptional activity upon FASN blockade. To test this hypothesis, we used three different *in vitro* breast cancer models: (a) ERα-positive BT-474 breast cancer cells that exhibit gene amplification and constitutive phosphorylation of HER2 and MAPK,^36^ (b) ERα-positive MCF-7 cells stably transfected with a full-length HER2 cDNA (MCF-7/HER2-18 cells), which exhibit 45-fold higher levels of HER2 protein relative to control cells and MAPK hyperactivation,^36^ and (c) ERα-positive MCF-7 cells stably infected with a full-length HRG cDNA (MCF-7/HRG cells), which exhibit persistent activation of HER2/3/4 receptors and MAPK relative to control cells.^38, 39^ Although the ability of E_2_ to activate ERE-luciferase transcription varied significantly among the three cell lines, both HER2- (Figure 4, left and middle panels) and HRG- (Figure 4, right panel) overexpressing breast cancer cells showed wild-type ERα function in response to exogenous ligand. Notably, pharmacological FASN blockade using sub-optimal concentrations of C75 greatly increased E_2_-dependent ERα activity in all three-breast cancer models. Accordingly, a dramatic ∼10-fold increase in E_2_-stimulated ERα-dependent transcriptional activity was observed in MCF-7/HER2-18 cells upon FASN inhibition (Figure 4, middle panel). Supporting the notion that this enhancement of E_2_ action was attributable to the activation of non-genomic ERα/MAPK cross-talk, treatment with U0126 decreased ERα transcriptional activity to the baseline response obtained in the presence of E_2_ alone. Furthermore, co-treatment with U0126 and the ERα antagonist ICI 182 780 abolished the extraordinarily high levels of ERE reporter activity obtained in BT-474, MCF-7/HER2-18 and MCF-7/HRG cells co-treated with E_2_ and C75.

### FASN inhibition blocks E_2_-stimulated breast cancer cell growth and survival

**Figure 5:**
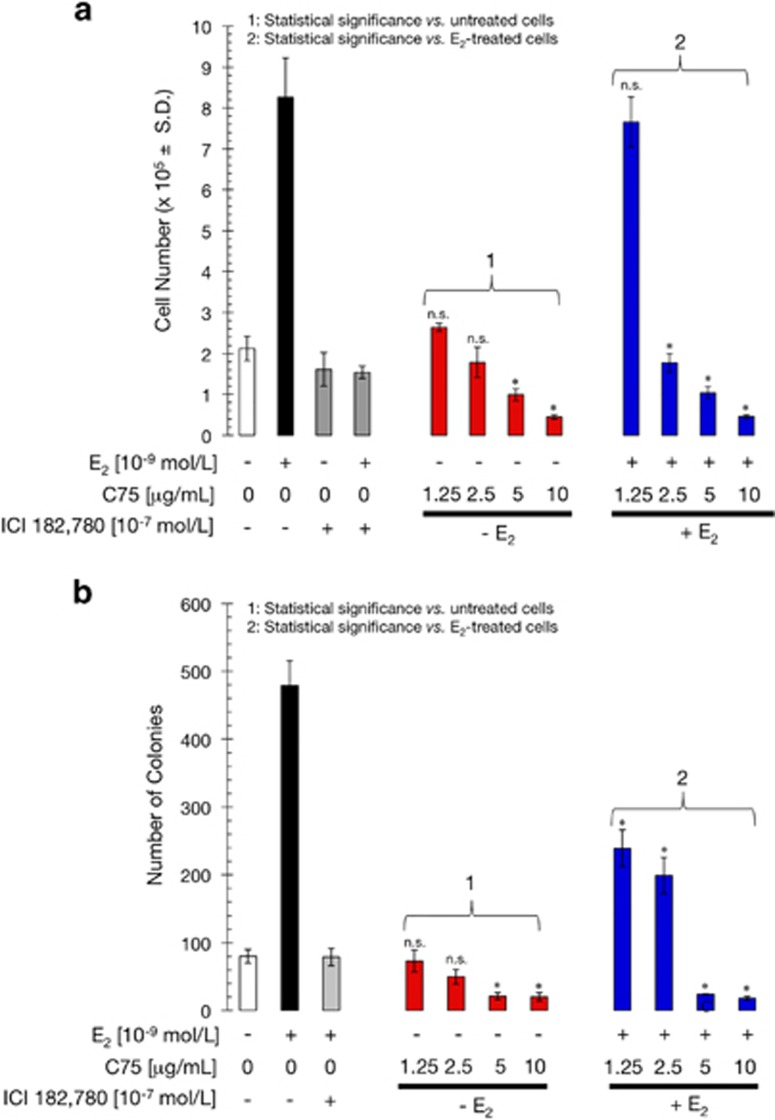
FASN inhibition blocks E_2_-stimulated cell growth and survival. (**a**) E_2_-depleted MCF-7 cells were plated in 24-well plates at 10 000 cells/well in phenol red-free IMEM and 5% CCS containing E_2_ (10^−9^ mol/l), ICI 182 780 (10^−7^ mol/l), C75 (1.25–10 μg/ml), or combinations of these compounds as specified, and ethanol (v/v) or DMSO (v/v) vehicles alone. The data presented are mean of number cells × 10^4^/well (columns)±s.d. (bars) after 6 days of treatment. All assays were performed at least three times in triplicate. (**b**) E_2_-depleted MCF-7 cells were plated in soft agarose containing E_2_ (10^−9^ mol/l), ICI 182 780 (10^−7^ mol/l), C75 (1.25–10 μg/ml), and ethanol (v/v) or DMSO (v/v) vehicles alone for 7–10 days. Colony formation (⩾50 μm) was assessed using a colony counter. Each experimental value represents the mean colony number (columns)±s.d. (bars) from at least three separate experiments in which triplicate dishes were counted. Non-significant (NS) differences (*P*>0.05) were identified by ANOVA followed by Scheffé's multiple contrasts; *P<0.05 compared with control cells by ANOVA followed by Scheffé's multiple contrasts. (1) Control cells in culture medium only. (2) Control cells in medium supplemented with 10^−9^ mol/l E_2_.

We next evaluated whether the transcriptional effects described above correlated with an active involvement of FASN signaling in E_2_-mediated breast cancer cell proliferation and survival. When MCF-7 cells were starved of E_2_ in medium containing charcoal-treated calf serum (CCS), replated and then grown in the absence or presence of 10^−9^ mol/l E_2_ for 5 days, we failed to observe any significant enhancement of the anchorage-dependent cell growth of E_2_-depleted MCF-7 cells in response to increasing concentrations of C75 (Figure 5a). Exogenous supplementation with E_2_ notably enhanced MCF-7 cell proliferation, which was prevented by ICI 182 780 (Figure 5a). Low concentrations of C75 (< 5 μg/ml) inhibited E_2_-enhanced MCF-7 cell growth; moreover, high concentrations of C75 (>5 μg/ml) decreased cell proliferation to below that observed in the absence of E_2_, strongly suggesting that FASN inhibition-induced hyperactivation of ERα-driven transcriptional activity does not promote cell proliferation but instead induces significant cytostatic and cytotoxic effects in E_2_-dependent breast cancer cells.

As the soft agar colony-forming assay measures both anchorage-independent cell proliferation and survival, and because it is well established that MCF-7 cells cannot growth independently of anchorage in the absence of E_2_, we next examined the effects of C75 on the clonogenic capacity of MCF-7 cells on soft agar. As single agent, C75 failed to stimulate the extremely low ability of MCF-7 cells to from colonies in semisolid media (Figure 5b). As expected, E_2_ treatment induced anchorage-independent growth of MCF-7 cells, which was blocked by ICI 182 780. Similar to ICI 182 780, C75 treatment was sufficient to prevent the E_2_-stimulated anchorage-independent cell growth of MCF-7 cells (Figure 5b). We thus conclude that FASN inhibition-promoted hyperactivation of ERα signaling exerts strong antitumor effects in hormone-dependent breast cancer cells.

### FASN inhibition upregulates and promotes the nuclear accumulation of the E_2_ targets p21^WAF1/CIP1^ and p27^Kip1^ while downregulating the PI3K/AKT pro-survival pathway

**Figure 6:**
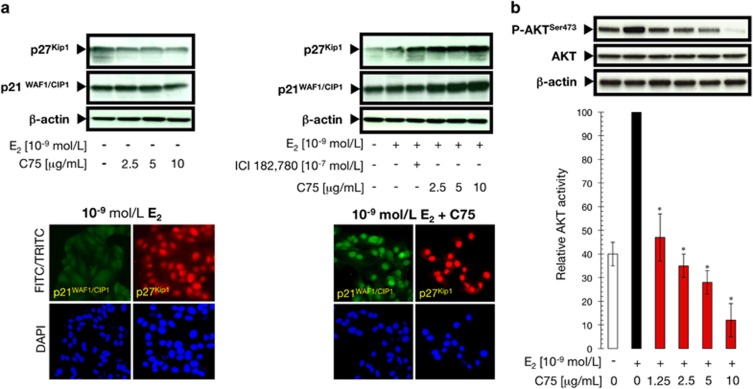
FASN inhibition activates p21^WAF1/CIP1^ and p27^Kip1^ and simultaneously deactivates the PI3K/AKT pathway. (**a**) Left: Protein was isolated from MCF-7 cells following treatment with C75 for 48 h in the absence or presence of 10^−9^ mol/l E_2_, as described in ‘Materials and methods'. p21^WAF1/CIP1^ and p27^Kip1^ expression was determined by immunoblotting using anti-p21^WAF1/CIP1^ mouse monoclonal and anti-p27^Kip1^ rabbit polyclonal antibodies, respectively. The blot was stripped and re-probed with an antibody to β-actin to assess equal loading of lysate proteins and transfer. Figure shows a representative immunoblot analysis. Similar results were obtained in three independent experiments. Right: E_2_-depleted MCF-7 cells were seeded at 1 × 10^4^ cells/well in a four-well chamber slide. After 48 h incubation with 10^−9^ mol/l E_2_ in the absence or presence of 5 μg/ml C75, p21^WAF1/CIP1^ and p27^Kip1^ cellular localization was evaluated after a 2 h incubation with anti-p21^WAF1/CIP1^ mouse monoclonal and p27^Kip1^ rabbit polyclonal antibodies diluted 1:200 in 0.05% Triton X-100/PBS. After labeling, cells were extensively washed, and the localization of p21^WAF1/CIP1^ and p27^Kip1^ was detected by indirect immunofluorescence by incubating with FITC-conjugated anti-mouse (p21^WAF1/CIP1^) or TRITC-conjugated anti-rabbit (p27^Kip1^) IgG secondary antibodies. Figure shows a representative immunostaining analysis. Similar results were obtained in three independent experiments. (**b**) Protein was isolated from E_2_-stimulated MCF-7 cells following treatment with C75 for 6 h as described in ‘Materials and methods', and AKT activation was determined by immunoblotting using a specific phospho-AKT polyclonal antibody. The blot was stripped and re-probed with antibodies for total AKT protein and β-actin to assess equal loading of lysate proteins and transfer. Bottom: Immunoreactive bands for phospho-AKT were scanned and normalized to total AKT. After a second normalization to β-actin, the value obtained in E_2_-stimulated MCF-7 cells was set to 100% and the different treatment groups were expressed as a percentage of control levels. Data are presented as the mean±s.d. of two independent experiments and the asterisks indicate values that are significantly different (*P<0.05) from E_2_-stimulated AKT activity.

To further explore the molecular mechanisms through which FASN blockade might interrupt E_2_-dependent mitogenic ERα signaling, we first evaluated the effects of C75 on the expression of p21^WAF1/CIP1^ and p27^Kip1^ CDK inhibitors (CDKIs), two key players in the E_2_-induced regulation of cell cycle progression and critical mediators of the therapeutic effects of antiestrogen in breast cancer.^40, 41, 42, 43, 44^ The expression of p21^WAF1/CIP1^ and p27^Kip1^ proteins after E_2_ depletion were not drastically altered by C75 treatment (Figure 6a). A modest increase in p21^WAF1/CIP1^ and p27^Kip1^ expression was observed after treatment of E_2_-stimulated MCF-7 cells with ICI 182 780, thus corroborating the role of these CDKIs as critical mediators of the growth inhibitory effects of antiestrogens in breast cancer cells.^43, 44^ Remarkably, p21^WAF1/CIP1^ and p27^Kip1^ expression was substantially increased in E_2_-stimulated MCF-7 cells treated with increasing concentrations of C75 (Figure 6a). Moreover, immunofluorescence analyses revealed that FASN inhibition resulted in the almost complete translocation of p21^WAF1/CIP1^ and p27^Kip1^ from the cytosol to the cell nucleus (Figure 6a).

**Figure 7:**
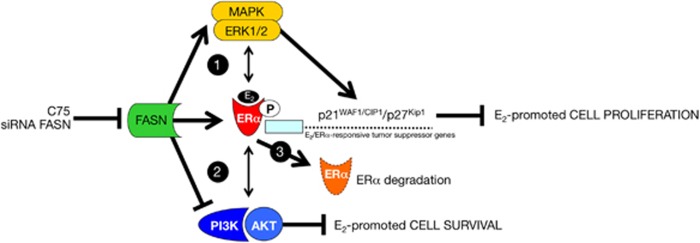
FASN-regulated ERα signaling in breast cancer cells: a working model. Scheme showing a hypothetical model for FASN inhibition-induced regulation of E_2_/ERα signaling in breast cancer. MAPK/ERK and PI3K/AKT non-genomic cross-talk with E_2_-activated ERα signaling mediates a variety of cellular responses including proliferation, survival, cell cycle arrest and apoptosis. Although activation of the MAPK pathway is generally associated with increased cell proliferation, an increasing number of studies have shown that prolonged MAPK/ERK activation can induce the expression of CDKIs, such as p21^WAF1/CIP1^ and p27^Kip1^, leading to growth inhibition. Phosphorylation of ERα by kinases such as ERK1/2 significantly enhances the transcriptional activity of ERα, a known transcriptional activator of p21^WAF1/CIP1^ and p27^Kip1^. The ability of C75-induced FASN inhibition to activate non-genomic ERα/MAPK cross-talk might therefore synergistically activate ERα-regulated tumor suppressive responses to block E_2_-promoted cell proliferation (1). C75-induced FASN inhibition simultaneously leads to the suppression of AKT activity, another non-genomic cross-talk mechanism with ERα, underlying the E_2_-stimulated anchorage-independent growth of breast cancer cells (2), meanwhile, C75-induced FASN blockade also causes ERα knockdown likely via degradation through the ubiquitination-proteasome pathway and/or inhibition of ERα gene expression (3).

The PI3K signaling pathway together with its downstream target AKT is thought to have important roles not only in the transcriptional activation mediated by ERα but also in ERα-promoted cell proliferation and anti-apoptotic responses, including those necessary for anchorage-independent growth and survival in soft agar.^45, 46, 47, 48, 49^ We therefore monitored the phosphorylation status of AKT at Ser^473^ in MCF-7 cells following exposure to E_2_ and C75. Interestingly, the high levels of phospho-AKT^Ser473^ achieved upon stimulation of MCF-7 cells with E_2_ were downregulated in the presence of graded concentrations of C75 (>75% decrease), whereas the levels of total AKT remained unchanged (Figure 6b). Concomitantly, FASN inhibition augmented the E_2_-mediated downregulation of ERα protein (Supplementary Figure 2), strongly suggesting that FASN signaling regulates ERα signaling by functionally synergizing (MAPK/ERK) or antagonizing (PI3K/AKT) key molecular components of non-genomic ERα cross-talk to negatively regulate E_2_-stimulated breast cancer cell growth and survival while promoting the reduction of ERα protein (Figure 7).

## Discussion

We herein report a previously unrecognized molecular interaction between endogenous fatty acid metabolism and ERα signaling in human breast cancer cells that is complex and seems to occur at multiple levels. We demonstrate that perturbation of the lipogenic activity of FASN using the slow-binding inhibitor of FASN activity, C75, markedly enhances E_2_-stimulated ERα transactivation in ERE reporter assays. Additionally, our data indicate that specific depletion of FASN gene activity dramatically decreases E_2_ requirements for optimal transactivation of ERα, further confirming the specific ability of FASN signaling to regulate the degree of sensitivity of breast cancer cells to E_2_-stimulated ERα-driven transcriptional activity.

FASN inhibition/depletion does not promote E_2_-independent ERα-driven transcriptional activity. However, the synergistic interaction observed between C75 and E_2_ strongly suggests that FASN perturbation alters ERα activity by regulating signaling pathways that converge on ERα itself. ERα cross-talks with a number of signaling pathways such as MAPK, and these molecular interactions may enhance E_2_-dependent ERα transactivation. Therefore, the striking ability of C75-induced FASN blockade to augment E_2_-induced ERα transactivation can be explained as follows. FASN inhibition triggers non-genomic, E_2_-dependent, ERα/MAPK cross-talk that mechanistically participates in the hypersensitivity to E_2_. Accordingly, MAPK activity is enhanced following co-exposure to E_2_ and the FASN inhibitor C75 or RNAi against FASN. Upon co-administration of the MAPK inhibitor U0126, the synergistic effect of FASN inhibition on E_2_-stimulated ERα transactivation is abolished and reverts to the baseline response obtained in the presence of E_2_ alone. But, MAPK activation is not solely responsible for E_2_-dependent ERα transactivation because blockade of this enzyme does not completely abrogate E_2_ hypersensitivity. Interestingly, in the presence of the FASN inhibitor C75, the ERα antagonist ICI 182 780, and the MAPK inhibitor U0126, E_2_-stimulated ERα-driven transcriptional activity reverts to the basal level seen in E_2_-depleted breast cancer cells. This observation confirms that the ability of FASN inhibition to enhance the genomic activity of ERα depends on the upstream involvement of an E_2_/ERα-triggered MAPK signaling cascade.^50, 51^ Indeed, our data support a model in which FASN inhibition triggers hyperactivation of an ERα-regulated *Ras/Raf*/MEK1/MEK2 → ERK1/ERK2 MAPK signaling pathway without promoting ligand (E_2_)-independent activation of ERα transactivation. Although the ultimate mechanisms connecting FASN, MAPK, and ERα remain to be elucidated, the proposed mechanism of action for FASN inhibitors links high levels of intracellular malonyl-CoA upon FASN inhibition to potential downstream effects.^52, 53, 54, 55^ Interestingly, we did not observe any changes in the activity of MAPK or ERα upon treating cells with bezafibrate, a specific inhibitor of acetyl-CoA carboxylase,^56^ the rate-limiting enzyme of the fatty acid synthesis pathway (data not shown). Therefore, supra-physiological cytosolic accumulation of the FASN substrate malonyl-CoA, a key metabolite in the regulation of energy homeostasis,^57^ rather than depletion of the FASN end-product palmitate, may provide a molecular bridge linking FASN-dependent endogenous fatty acid metabolism, the MEK1/2 → ERK1/2 signaling pathway, and ERα activity in breast cancer cells. In this regard, a link between sex steroid receptors and fatty acid metabolism has been established through alterations in FASN expression and malonyl-CoA levels,^58, 59, 60^ thus making plausible the notion that malonyl-CoA can transmit the metabolic stress imposed by FASN inhibition to ERα activity and E_2_-responsive gene expression in breast cancer cells. Because ERα interacts with the α-catalytic subunit of the central energy sensor AMP-activated protein kinase (AMPK),^61^ whereas the substrate accumulation but not end-product depletion of fatty acids from FASN inhibition triggers AMPK activation,^62, 63^ our findings might implicate *de novo* lipogenesis operating as a *bona fide* metabolic signal transmitter,^64^ regulating breast cancer cell sensitivity not only to E_2_ but also to antiestrogen therapies.^65, 66^

Our results suggest a novel mechanism through which pharmacological blockade of FASN-catalyzed endogenous lipogenesis might promote an ERα-related suppression of E_2_-stimulated breast cancer cell growth and survival. First, as pure nonsteroidal antiestrogens have been found to antagonize the proliferative activity of E_2_ by promoting the upregulation of p21^WAF1/CIP1^ and p27^Kip1^ expression and their nuclear recruitment into cyclin E-Cdk2 complexes,^43, 44^ it appears reasonable to suggest that an analogous p21^WAF1/CIP1/^p27^Kip1^-dependent cell growth-arresting mechanism might occur in response to C75-induced ablation of FASN signaling in E_2_-dependent breast cancer cells. Thus, because FASN inhibition apparently increases the amount of p21^WAF1/CIP1^ and p27^Kip1^ available for binding to and inhibiting Cdk2 activity, FASN inhibitors such as C75 could operate as a *bona fide* antiestrogen suppressing E_2_-promoted cell cycle. Second, C75-induced FASN blockade was found to significantly prevent, to a similar extent, E_2_-stimulated AKT activity and E_2_-stimulated anchorage-independent growth of breast cancer cells, which has been shown to be mediated by non-genomic cross-talk between ERα and the PI3K/AKT pathway.^45^ Therefore, the interruption of AKT signaling might have a key role in determining the antiestrogenic actions that were observed following C75-induced blockade of FASN activity. Third, analogous to other pure antiestrogens, C75 treatment resulted in ERα protein downregulation while apparently promoting MAPK activation and ERα transactivation. Considering previous studies showing that activation of MAPK/ERK signaling can induce p21^WAF1/CIP1^ expression and that ERα is a known transcriptional regulator of p21^WAF1/CIP1^,^67, 68, 69^ additional studies are warranted to investigate how MAPK/ERK and ERα might synergistically regulate C75-induced p21^WAF1/CIP1^ expression. Although the precise mechanism of C75-mediated reduction of ERα protein remains unclear, it is plausible that loss of FASN signaling induces proteasome-dependent ERα degradation and/or epigenetic inhibition of ERα mRNA synthesis, driving target cells to completely different outcomes to those induced when E_2_ binding acts as the natural signal for ERα degradation and the parallel reduction in ERα mRNA levels.^70, 71^

In summary, our findings reveal an unanticipated ability of FASN-catalyzed endogenous lipogenesis to modulate the sensitivity of breast cancer cells to E_2_-dependent ERα signaling *via* regulation of non-genomic MAPK/ERα and AKT/ERα cross-talk. FASN inhibition can mediate opposing effects on the E_2_/ERα-related conflicting cellular responses driven by the MAPK/ERK and PI3K/AKT pathways by switching the balance toward MAPK/ERK activation, inducing cell cycle inhibiting genes such as p21^WAF1/CIP1^ and p27^Kip1^, while suppressing PI3K/AKT-mediated cell cycle progression and cell survival, synergistically leading to suppression of E_2_-promoted anchorage-dependent and -independent cell growth in hormone-dependent breast cancer cells. Previously, we observed that C75-induced FASN inhibition works as a potent antagonist of E_2_- and tamoxifen-dependent ERα-driven transcriptional activation in human endometrial adenocarcinoma cells.^72^ We now demonstrate that C75-regulated MAPK/ERK signaling activation, PI3K/AKT pathway inhibition, and ERα signaling work together to promote disruption of human breast cancer cell growth and survival. Because new generations of FASN inhibitors have recently entered the clinic,^73, 74^ this novel mechanism might represent a promising strategy for future FASN-based clinical trials for hormone receptor-positive breast cancer.

## Materials and methods

### Materials

Phenol red-free Improved Minimal Essential Medium (IMEM) was purchased from Biofluids (Rockville, MD, USA); dextran-coated, charcoal-treated bovine serum (CCS) from Biosource International (Camarillo, CA, USA); E_2_ from Sigma Chemical Co. (St Louis, MO, USA), and ICI 182 780 was a gift from Zeneca Pharmaceuticals. C75 was purchased from Alexis Biochemicals (San Diego, CA, USA), dissolved in DMSO, and stored as a stock solution (25 mg/ml) at −20 °C in the dark. The primary antibodies used in this studies were obtained from the following suppliers: FASN monoclonal antibody (clone 23) was from BD Biosciences Pharmingen (San Diego, CA, USA); β-actin goat polyclonal, ERα (G-20) rabbit polyclonal antibody, and p27^KIP1^ rabbit polyclonal antibodies were from Santa Cruz Biotechnology (Santa Cruz, CA, USA). ERK1/2, AKT, phospho-ERK1/2, and phospho-AKT^Ser473^ rabbit polyclonal antibodies were from Cell Signaling Technology (Beverly, MA, USA). p21^WAF1/CIP^ mouse monoclonal antibody was from PharMingen.

### Cell lines and culture conditions

MCF-7 and BT-474 breast cancer cells were grown in IMEM containing 5% (v/v)-heat-inactivated fetal bovine serum and 2 mmol/l l-glutamine. Cells were maintained at 37 °C in a humidified atmosphere of 95% air and 5% CO_2_. The characterization of and growth conditions for MDA-MB-231 breast cancer cells stably transfected with wild-type ERα (S30 cells) have been reported previously.^22, 23^ MCF-7 cells stably overexpressing the HER2 oncogene (MCF-7/HER2-18) were kindly provided by Dr Mien-Chie Hung (University of Texas M.D. Anderson Cancer Center, Houston, TX, USA).

HRG-overexpressing MCF-7 cells (MCF-7/HRG) were engineered by infecting MCF-7 cells with the retroviral vector pBABE-puromycin containing the full-length cDNA of HRG-β2 generated by PCR using the HRG-β_2_ cDNA accession number 183996 as a template. The PCR product was cloned into the retroviral expression vector pBABE-puromycin using *Bam*HI and *Eco*RI restriction sites and transfected into a high-efficiency transient packaging system using FuGENE 6 reagent (Roche Biochemicals, Indianapolis, IN, USA). Medium from transfected cells was collected after 48 h, filtered, and used to infect MCF-7 cells for 24 h in the presence of polybrene (Sigma). Infected MCF-7 cells were grown for an additional 24 h in standard medium, and stable cell lines (MCF-7/HRG and matched control MCF-7/pBABE cells) were selected and expanded in the presence of 2.5 μg/ml puromycin for at least 2 weeks. All cell lines were free of *Mycoplasma* contamination and were not re-authenticated.

### ERα transcriptional activity

Cells were propagated in E_2_-deprived IMEM with 5% CCS for 5 days before initiating experiments. Cells were seeded in 12-well plates (1 × 10^5^ cells/well) and co-transfected as above with 1 mg/well of the estrogen-responsive reporter, ERE-Luc, containing a *Xenopus* vitellogenin A_2_-derived ERE, and 0.1 μg/well of the internal control plasmid pRL-CMV to correct for transfection efficiency. After 18 h, cells were washed and incubated in fresh medium containing 5% CCS supplemented with E_2_ (10^−9^ M), ICI 182 780 (10^−7^ M), C75 (1.25–10 μg/ml), U0126 (20 μmol/l), combinations of these compounds as specified, or ethanol (v/v) or DMSO (v/v) vehicle alone. At 24 h post treatment, luciferase activity from cell extracts was detected using a Luciferase Assay System (Promega, Madison, WI, USA) and a TD-20/20 luminometer (Turner Designs, Sunnyvale, CA, USA). The magnitude of activation in ERE-luciferase-transfected cells treated with vehicle alone was determined after normalization to the activity of pRL-CMV and was considered 1.0. This control value was used to calculate the relative (fold) change in transcriptional activities of ERE-luciferase-transfected cells in response to treatment, after normalization to pRL-CMV activity. All data were normalized as the ratio of raw light units to pRL-CMV units corrected for pRL-CMV activity.

### RNAi-mediated silencing of FASN

Synthetic oligonucleotides targeting FASN were purchased from Dharmacon RNA Technologies (Lafayette, CO, USA). The double-stranded siRNA sequences were as follows: sense CCCUGAGAUCCCAGCGCUGdTdT and antisense, CAGCGCUGGGAUCUCAGGGdTdT. The design of these siRNAs was based on a DNA sequence of the type AA(N_19_) corresponding to nucleotides 1210–1231 located 3′ to the first nucleotide of the start codon of the human FASN cDNA (AACCCTGAGATCCCAGCGCTG). Searches of the human genome database (BLAST) were conducted to ensure that the sequences would not target other gene transcripts. Transfections were performed in 60-mm dishes at a density of 0.4–0.5 × 10^6^ cells/dish using FuGENE 6 with the final concentration of anti-FASN siRNA of 100 and 200 nmol/l. As a nonspecific siRNA control, cells were transfected with equimolar concentrations of a Non-Specific Control Pool (siRNA negative control; Upstate Cell Signaling Solutions-Dharmacon RNA Technologies; Catalog #D-001206-13). At the indicated times after transfection, cells were used for FASN activity assays, immunoblotting, or assays for ER-driven reporter activity.

### FASN activity

FASN activity was assayed in particle-free supernatants by recording the decrease of A_340_ nm due to oxidation of NADPH at 25 °C, essentially as described by Dils and Carey.^21^

### Anchorage-dependent growth assays

Cells were grown in phenol red-free IMEM and 5% CCS for 5 days in T75 flasks. Cells were trypsinized and re-plated in 24-well plates at 10 000 cells/well. Cells were incubated for 18 h to allow attachment, after which a zero time point was determined. Cells were treated with phenol red-free IMEM and 5% CCS containing either E_2_ (10^−9^ mol/l), ICI 182 780 (10^−7^ mol/l), C75 (1.25–10 μg/ml), or combinations of these compounds as specified. Cells were counted at day 0, 3, and 6 with a Coulter Counter (Coulter Electronics, Hialeah, FL, USA). All assays were performed at least three times in triplicate. The data are presented as mean number of cells × 10^4^/well±s.d.

### Soft agar colony formation assays

Cells were grown in phenol red-free IMEM and 5% CCS for 5 days in T75 flasks. A bottom layer of 1 ml IMEM containing 0.6% agar and 10% CCS was prepared in 35 mm multi-well cluster dishes. After the bottom layer solidified, cells (20 000/dish) were added in a 1 ml top layer containing E_2_, ICI 182 780, C75 or combinations of these compounds as specified, ethanol (v/v), or DMSO (v/v), and 10% CCS. All samples were prepared in triplicate. Dishes were incubated in a humidified 5% CO_2_ incubator at 37 °C and colonies measuring ⩾50 μm were counted after ∼14 days with a cell colony counter after staining with nitroblue tetrazolium (Sigma).

### Immunoblotting

Cells were washed two times with PBS and then lysed in buffer (20 mmol/l Tris (pH 7.5), 150 mmol/l NaCl, 1 mmol/l EDTA, 1 mmol/l EGTA, 1% Triton X-100, 2.5 mmol/l sodium pyrophosphate, 1 mmol/l β-glycerolphosphate, 1 mmol/l Na_3_VO_4_, 1 g/ml leupeptin, 1 mmol/l phenylmethylsulfonylfluoride) for 30 min on ice. The lysates were cleared by centrifugation (15 min at 14 000 r.p.m., 4 °C). Protein content was determined against a standardized control using the Pierce protein assay kit (Rockford, IL, USA). Equal amounts of protein were heated in SDS sample buffer (Laemmli) for 10 min at 70 °C, subjected to electrophoresis on 3-8% Tris-Acetate NuPAGE or 10% SDS–PAGE, and transferred to nitrocellulose membranes. For immunoblot analyses of p21^WAF1/CIP1^, p27^KIP1^, and ERα, nonspecific binding on the nitrocellulose filter was minimized by blocking for 1 h at room temperature (RT) with TBS-T (25 mmol/l Tris-HCl, 150 mmol/l NaCl (pH 7.5), and 0.05% Tween 20) containing 5% (w/v) nonfat dry milk. The treated filters were washed in TBS-T and then incubated with primary antibodies for 2 h at RT in TBS-T containing 1% (w/v) nonfat dry milk. The membranes were washed in TBS-T, horseradish peroxidase-conjugated secondary antibodies (Jackson Immuno Research, West Grove, PA, USA) in TBS-T were added for 1 h, and immunoreactive bands were visualized with the ECL detection reagent (Pierce). For immunoblot analyses of AKT and phospho-AKT^Ser473^, membranes were blocked as described and incubated overnight at 4 °C with primary antibody in TBS-T/5% bovine serum albumin (BSA). The membranes were washed in TBS-T, horseradish peroxidase-conjugated secondary antibodies in TBS-T containing 5% (w/v) nonfat dry milk were added for 1 h, and primary antibody binding was detected as described. Blots were re-probed with an antibody to β-actin to control for protein loading and transfer. Densitometric values of protein bands were quantified using Scion imaging software (Scion, Frederick, MD, USA).

### *In situ* immunofluorescent staining

Cells were seeded at 1 × 10^4^ cells/well in a four-well chamber slide (Nalge Nunc International, Rochester, NY, USA). After a 48 h incubation with C75, cells were washed with PBS, fixed with 4% paraformaldehyde in PBS for 10 min, permeabilized with 0.2% Triton X-100/PBS for 15 min, and stored overnight at 4 °C with 10% horse serum in PBS. The cells were washed and then incubated for 2 h with anti-p21^WAF1/CIP1^ mouse monoclonal or anti-p27^Kip1^ polyclonal antibodies diluted 1:200 in 0.05% Triton X-100/PBS. After extensive washing, the cells were incubated for 45 min with fluorescein isothiocyanate (FITC)-conjugated anti-mouse IgG, tetramethylrhodamine isothiocyanate (TRIC)-conjugated anti-rabbit IgG, or FITC-conjugated anti-rabbit IgG secondary antibodies (Jackson Immunoresearch Labs, West Grove, PA, USA) diluted 1:200 in 0.05% Triton X-100/PBS. The cells were washed five times with PBS and mounted with VECTASHIELD+DAPI (Vector Laboratories, Burlingame, CA, USA). As controls, cells were stained with primary or secondary antibody alone. Controls did not display significant fluorescence in any case (data not shown). Indirect immunofluorescence was recorded on a Zeiss microscope. Images were noise-filtered, corrected for background, and prepared using Adobe Photoshop (Adobes Systems, San Jose, CA, USA).

### Statistical analysis

All statistical analyses were performed using XLSTAT 2010 (Addinsoft). For all experiments, at least three independent experiments were performed with *n*⩾3 replicate samples per experiment. No statistical method was used to predetermine sample size. Investigators were not blinded to data allocation. Experiments were not randomized. All observations were confirmed by at least three independent experiments. Data are presented as mean±s.d. Comparisons of means of ⩾3 groups were performed by ANOVA, and the existence of individual differences, in case of significant *F* values at ANOVA, were tested by Scheffé's multiple contrasts. In all studies, *P*-values<0.05 were considered to be statistically significant (denoted as *). All statistical tests were two-sided.
