# Supplementary material for: Fatty acid synthase regulates estrogen receptor-α signaling in breast cancer cells
Source: Oncogenesis. 2017 Feb 27;6(2):e299–. doi: 10.1038/oncsis.2017.4 (PMC5337623; doi:10.1038/oncsis.2017.4)
Supplement: Supplementary Figure Legends [file oncsis20174x1.docx]

**SUPPLEMENTAL FIGURE LEGENDS**

**Supplemental Figure 1.** E_2_-depleted MCF-7 cells were transfected with double-stranded siRNA targeting FASN. At 72 h post-transfection, FASN expression was assessed by immunoblotting using an anti-FASN antibody (*top*) or by FASN activity assay (*bottom*). Data represent mean ± S.D. (n=3). n. s. Non-significant differences (*P* > 0.05) were identified by ANOVA followed by Scheffé’s multiple contrasts; ^*^ *P* < 0.05 compared with control cells by ANOVA followed by Scheffé's multiple contrasts

**Supplemental Figure 2.** E_2_-depleted MCF-7 cells were treated with increasing concentrations of C75 in combination with ICI 182,780 in the absence (*left*) or presence (*right*) of E_2_. ERα protein expression was analyzed by western blot. Immunoreactive bands for ERα were scanned and normalized to β-actin. Control values were set to 1, and the different treatment groups were expressed as a percentage of control levels.
