# Supplementary figures and images for: Fatty acid synthase regulates estrogen receptor-α signaling in breast cancer cells
Source: Oncogenesis. 2017 Feb 27;6(2):e299–. doi: 10.1038/oncsis.2017.4 (PMC5337623; doi:10.1038/oncsis.2017.4)

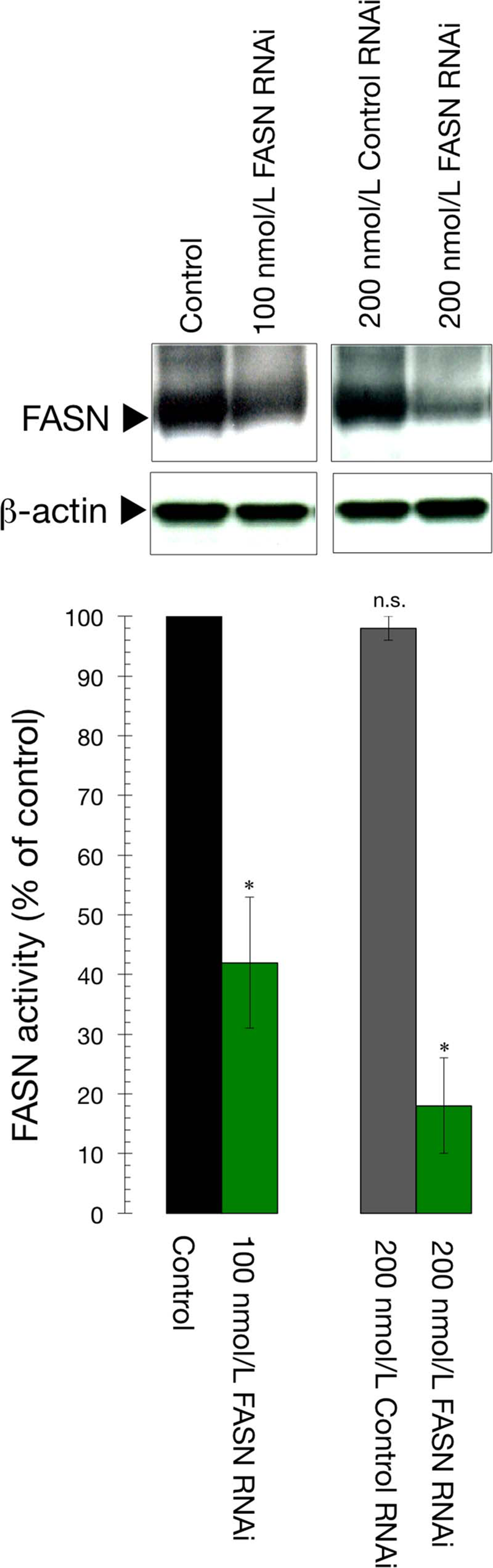

Supplement: Supplementary Figure 1 [file oncsis20174x2.tif]

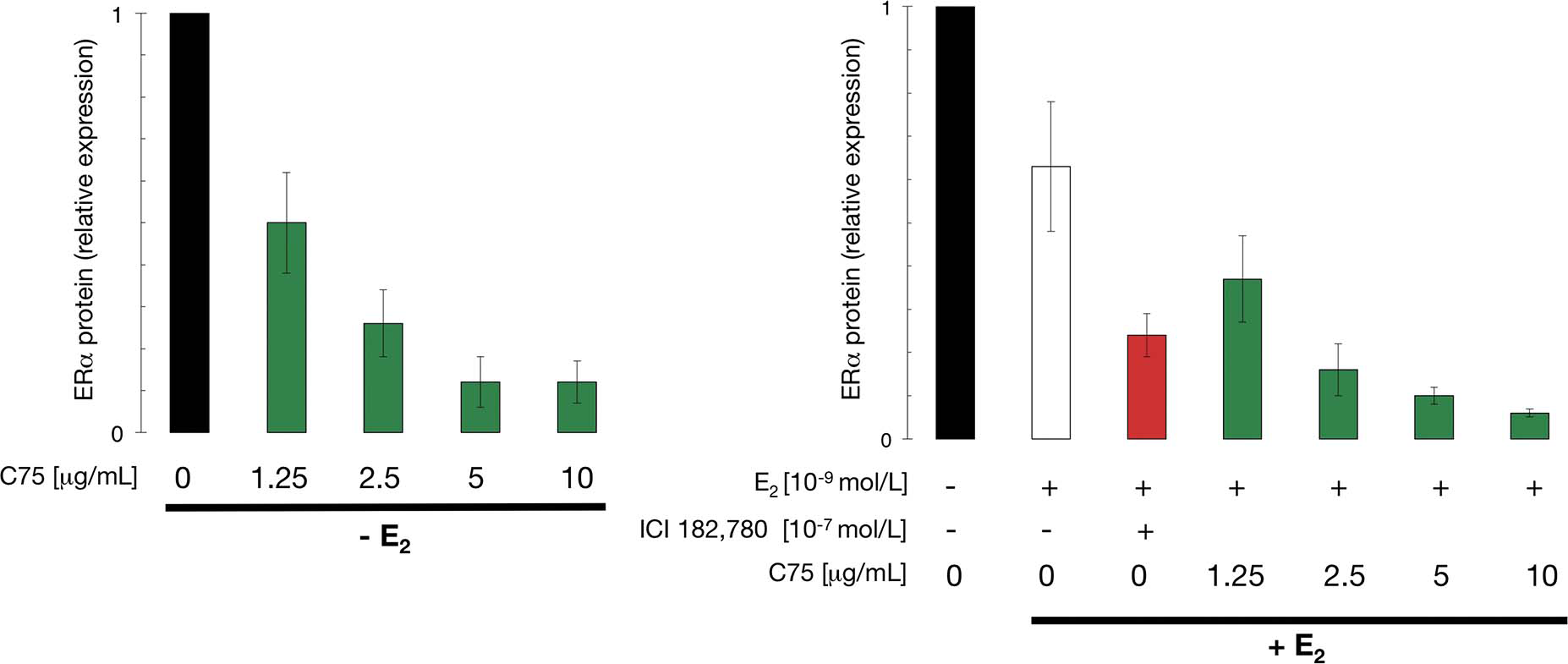

Supplement: Supplementary Figure 2 [file oncsis20174x3.tif]
